# Multivariate climate departures have outpaced univariate changes across global lands

**DOI:** 10.1038/s41598-020-60270-5

**Published:** 2020-03-03

**Authors:** John T. Abatzoglou, Solomon Z. Dobrowski, Sean A. Parks

**Affiliations:** 1 Department of Geography, University of Idaho, Moscow, 875 Perimeter Dr. Moscow, 83844 USA; 2 Management of Complex Systems Department, University of California, Merced, USA; 3 Department of Forest Management, University of Montana, Missoula, USA; 4 USDA Forest Service, Rocky Mountain Research Station, Aldo Leopold Wilderness Research Institute, Missoula, USA

**Keywords:** Climate change, Attribution, Climate-change ecology

## Abstract

Changes in individual climate variables have been widely documented over the past century. However, assessments that consider changes in the collective interaction amongst multiple climate variables are relevant for understanding climate impacts on ecological and human systems yet are less well documented than univariate changes. We calculate annual multivariate climate departures during 1958–2017 relative to a baseline 1958–1987 period that account for covariance among four variables important to Earth’s biota and associated systems: annual climatic water deficit, annual evapotranspiration, average minimum temperature of the coldest month, and average maximum temperature of the warmest month. Results show positive trends in multivariate climate departures that were nearly three times that of univariate climate departures across global lands. Annual multivariate climate departures exceeded two standard deviations over the past decade for approximately 30% of global lands. Positive trends in climate departures over the last six decades were found to be primarily the result of changes in mean climate conditions consistent with the modeled effects of anthropogenic climate change rather than changes in variability. These results highlight the increasing novelty of annual climatic conditions viewed through a multivariate lens and suggest that changes in multivariate climate departures have generally outpaced univariate departures in recent decades.

## Introduction

Climate change represents a key challenge to Earth’s biota. These changes will require understanding climate change impacts across multiple timescales and variables^1^. Place-based climate impacts to human-environment systems are often assessed with respect to local climate variability for a variable of interest (e.g., mean annual temperature), as systems are often adapted to such variability^2–4^. Individual climate attributes have already departed from historical ranges of variability in some regions, thereby contributing to climate impacts^5–9^. However, climate is intrinsically multivariate; many organisms and socio-ecological systems are adapted to and impacted by multiple climate variables and their interactions^10–15^. While climate change is typically portrayed by changes in individual climate variables, often considered independently of one another, changes in multivariate climatic conditions may be more appropriate for anticipating impacts to many systems.

Climate variables exhibit covariance on a variety of timescales through dynamic processes and land-surface interactions^16,17^. Covariance is particularly acute for water balance variables that are coupled through surface energy-water fluxes^10,18^ which are widely recognized as important factors controlling the distribution and productivity of agriculture and ecosystems, ecosystem disturbance, and water scarcity^19–22^. The covariance structure among climate variables results in combinations (e.g., warm and dry summers) that occur more frequently than if the variables were independent^23^. In contrast, variable combinations that are orthogonal to the canonical axis of variability (e.g., warm and wet summers) are more unusual in a climatic context and represent larger climate departures (*Supplemental Methods*). Covariance between variables is apparent on short time scales and contributes to meteorological extremes such as heat waves, and at longer timescales and contributes to climatological extremes such as droughts^16^. Herein, climate departures refer to absolute departures from a reference climate state in a univariate or multivariate sense. We argue that climate departures may be a valuable metric for tracking potential climate impacts that arise with anomalies of either sign from reference conditions^24,25^.

Here, we examine trends in multivariate annual climate departures for global land surfaces during 1958–2017 relative to 1958–1987 baseline conditions. We focus on four variables that encapsulate the climatic basis for Earth’s biota and have distinct impacts on human and natural systems: the coldest monthly average minimum temperature (T_n,min_), warmest monthly average maximum temperature (T_x,max_), annual actual evapotranspiration (AET), and annual climatic water deficit (D). These four variables collectively span thermal and moisture climatic axes and define the climatic niche of many species as well as climate impacts thereof. For instance, T_n,min_ influences the life history and distributions of species due to the physiological, ecological, and evolutionary impacts^26^ and is the basis of hardiness zones in the agricultural sector, while T_x,max_ has been shown to have impacts on electricity demand^27^ and crop yields^28^. AET and D are a reduced set of biologically relevant climate variables that represent the supply and unmet atmospheric demand components of the water balance^29^. AET is a proxy for productivity in natural systems^30^, while D is widely-used metric for ecosystem disturbance and the terrestrial carbon cycle^19,31^. In addition, various combinations of these variables are predictor variables for modeling species distributions^32,33^ and climate impacts^34,35^.

We use Mahalanobis distance and its standardization based on the Chi distribution^3,36^ to compute annual multivariate climate departures (σ_d_). The Mahalanobis distance compactly quantifies multiple variables, accounts for their covariance structure, and measures the distance in multivariate space away from a centroid through principal components analysis of standardized anomalies. σ_d_ can be interpreted as a multivariate z-score which scales climate departure with respect to local interannual climate variability. We compare σ_d_ to its univariate counterpart for individual variables using standardized Euclidean distance (e.g., σ_Tx,max_). To quantify the relative contribution of anthropogenic climate forcing to trends in climate departures, we develop a counterfactual simulation that removes the modeled climate change signal from observations. Two sensitivity experiments are used to elucidate the relative contribution of changes in means and changes in intra-annual to inter-annual variability on observed changes in climate departures. Both experiments remove the linear trends for variables during 1958–2017; the first experiment excludes linear trends in annual mean temperature, precipitation, and reference evapotranspiration, while the second only excludes linear trends in annual precipitation. Lastly, we compare observed trends in climate departures with those from internal climate variability using a 500 year control climate simulation.

## Results

**Figure 1:**
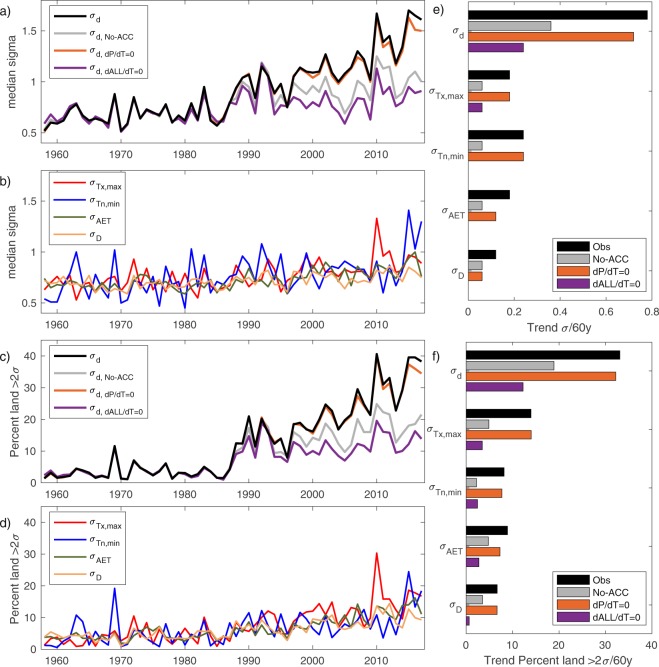
Annual time series and trends in median global climate departures. (**a**) Median annual multivariate climate departures (σ_d_), results from a counterfactual simulation that removed the modeled influence of anthropogenic climate change (σ_d,No-ACC_), and results from sensitivity experiments that removed the linear trend in annual precipitation (σ_d,dP/dT_) and annual precipitation, reference evapotranspiration, and temperature (σ_dALL/dT_). (**b**) Observed univariate climate departures for average maximum temperature of the warmest month (T_x,max_), average minimum temperature of the coldest month (T_n,min_), actual evapotranspiration (AET), and climatic water deficit (D). Annual percent of land surfaces with climate departures> 2σ for (**c**) σ_d_ and (**d**) individual variables. Global median sen-slope trends in (**e**) climate departures and (**f**) percent of land area>2σ during 1958–2017.

Positive trends in climate departures are evident in recent decades, particularly for σ_d_ (Fig. 1a,b). The four individual calendar years with the greatest global median σ_d_ coincided with the four warmest years globally over the period of analysis (2010, 2015–2017). The 60-year median trend in σ_d_ across global terrestrial surfaces was +0.78σ. Positive trends in climate departures over the 60-year period were observed for T_x,max_, T_n,min_, AET and D, but the magnitude of global median trends in σ_d_ was approximately three times greater than trends in climate departures for individual variables (Fig. 1e). Similarly, the geographic extent of land with σ_d_ exceeding 2 standard deviations increased markedly from a baseline of ~5% of land during the reference period (1958–1987) to ~30% during the most recent decade (2008–2017); increases in the geographic coverage of land >2 standard deviations for σ_d_ outpaced increases for individual variables (Fig. 1c,d,f). Similar results were seen using a truncated non-parametric approach for transforming individual variables, although the magnitude of trends was reduced due to the conservative approach (*Supplemental Methods*). In addition to observed trends, we found a weak correlation (r = 0.21, p = 0.1) between global median σ_d_ and the Multivariate ENSO Index^37^ averaged over Jan-Jun, highlighting the tendency for larger climate departures during El Niño years.

**Figure 2:**
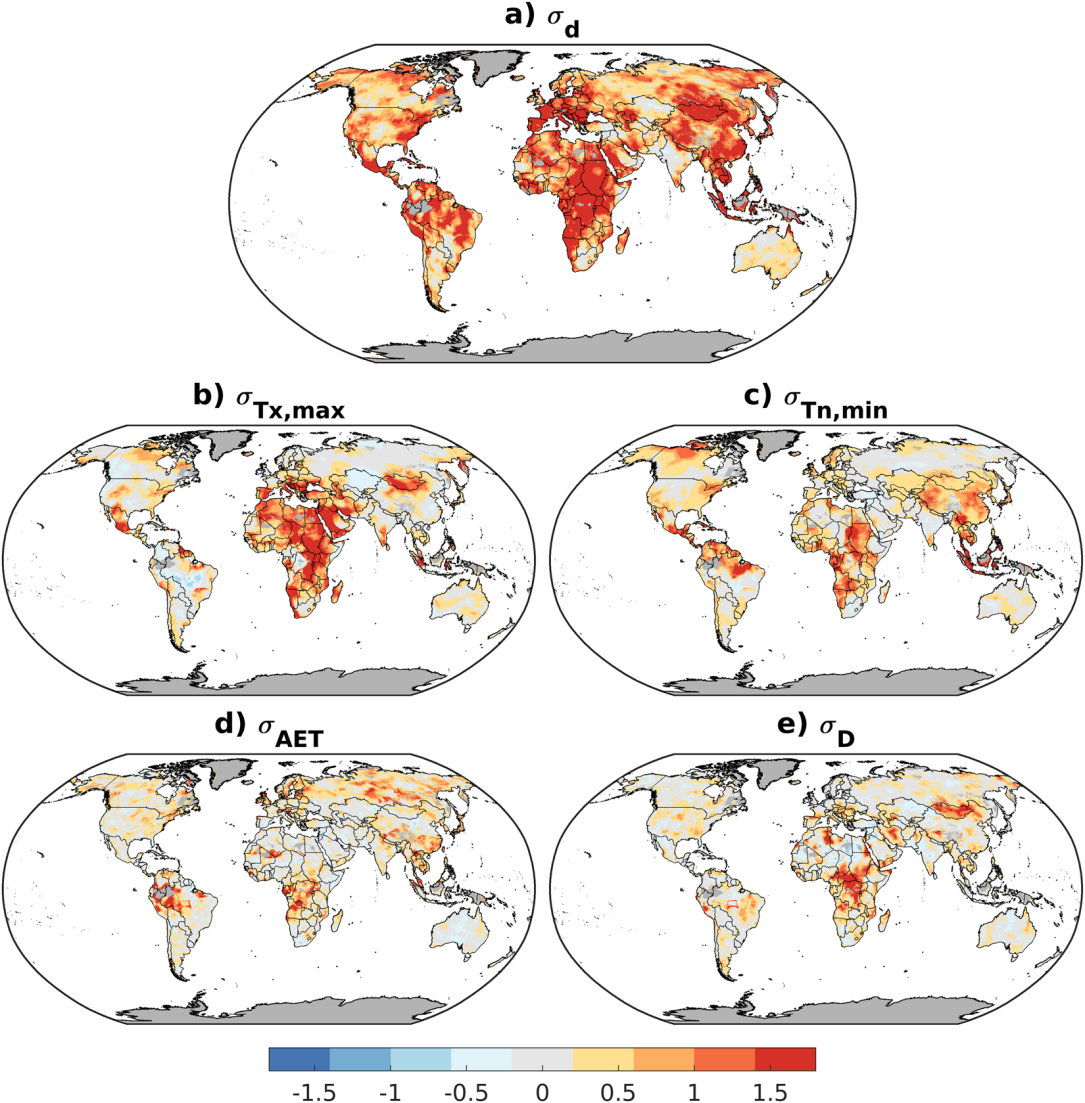
Trends in climate departures during 1958–2017. Linear trends (sen-slopes, 1958–2017) for (**a**) multivariate climate departure (*σ*_d_), (**b**) departure of warmest monthly maximum temperature (*σ*_Tx,max_), (**c**) departure of coldest monthly minimum temperature (*σ*_Tn,min_), (**d**) departure of actual evapotranspiration (*σ*_AET_), and (**e**) departure of climatic water deficit (*σ*_D_). Land areas where annual D or AET was 0 for a majority of the years in the baseline period are shown in grey.

Widespread positive trends in σ_d_ were observed over the past six decades across most terrestrial surfaces with statistically significant increases present for 58% of lands and the largest increases in southern Europe, southeast Asia, Africa, and the Amazon (Fig. 2a; Supplementary Fig. 1). Significant positive trends in σ_Tx,max_ and σ_Tn,min_ were seen across 16–18% of global lands (Fig. 2b,c). Significant positive trends in σ_AET_ were seen for 16% of global lands, primarily across high latitudes of the Northern Hemisphere and equatorial regions. Significant positive trends in σ_D_ were found for 11% of global lands. In contrast, significant negative trends in climate departures were confined to small geographic areas of 1–5% of land area depending on variable.

**Figure 3:**
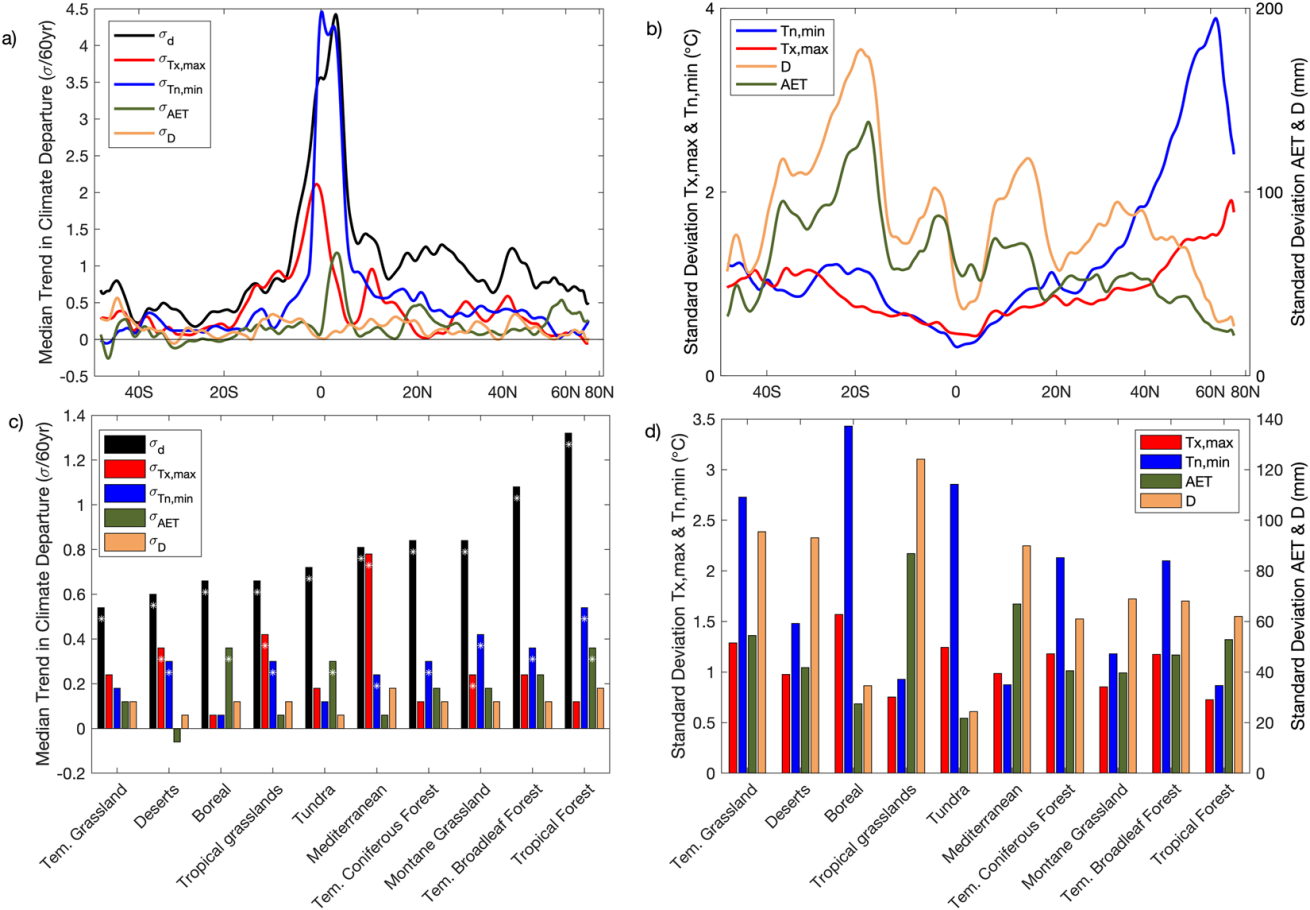
Latitudinal and biome based trends in climate departure. (**a**) Observed trends in median climate departures by latitude smoothed using a 1-degree moving mean. Panel (b) shows median standard deviation during 1958–1987 for the four individual climate variables by latitude, smoothed using a 1-degree moving window. (**c**) Observed trends in climate departures by biome ordered from the smallest to largest increase in σ_d_. Bars with asterisk indicate that at least 75% of the land area within each ecoregion had positive trends. Panel (d) shows median standard deviation for each of the climate variables by biome during 1958–1987.

Geographic hotspots of large positive trends often occurred in areas with low interannual variability. This is further demonstrated by examining trends in climate departures and the magnitude of interannual variability by latitude and biome. Latitudinal patterns show large positive trends in σ_d_ and σ_Tn,min_ near the equator co-located with regions with low variability during the reference period (Fig. 3a). Additionally, larger positive σ_d_ trends were seen in the Northern Hemisphere than the Southern Hemisphere (Fig. 3a). Trends for temperature-based departures generally exceeded moisture-based departures from 40°S to 50°N. At higher-latitudes, trends in σ_AET_ outpaced trends in temperature departures consistent with higher temperature variability at higher latitudes (Fig. 3b). Trends in climate departures by biome were generally largest where interannual variability among the four climate variables was low (Fig. 3c,d), with the largest positive σ_d_ trend in the Tropical Forest biome and the smallest positive trend in the Temperate Grassland biome.

Larger positive σ_d_ trends compared to univariate climate departure trends can be understood by considering the covariance structure and trends in the underlying climate variables. AET and D exhibited strong negative correlations on interannual timescales across most terrestrial surfaces, with T_x,max_ exhibiting positive correlation to D (Supplementary Fig. 3). By contrast, T_n,min_ was weakly correlated to the other climate variables, consistent with T_n,min_ being seasonally out of phase with the primary climatic factors that influence surface water availability. The principal components (PC) used in the Mahalanobis distance calculation largely reflect these relationships with the first loading projecting onto patterns of water limitations, loadings 2 and 3 projecting onto orthogonal dimensions related to T_n,min_ and T_x,max_, respectively, and loading 4 projecting to same signed anomalies of AET and D (Supplementary Fig. 4). Positive trends in T_x,max_ and T_n,min_ were seen across most terrestrial surfaces with AET and D trends showing more spatial variability but generally positive trends during 1958–2017 (Supplementary Fig. 5). Trends in σ_d_, by definition, arise from trends in Euclidean distances of PC scores. While no single loading predominantly accounted for σ_d_ trends, trends in PC4 and PC3 accounted for a majority of positive σ_d_ trends for 22% and 16% of the globe, respectively (Supplementary Table 1). Positive trends in AET and D were jointly observed for 46% of terrestrial surfaces that map onto the PC4 loading. Likewise, joint significant increases in T_x,max_ and T_n,min_ were observed for 24% of the globe and contributed to the positive trend in σ_d_ as T_x,max_ and T_n,min_ were poorly correlated on interannual timescales during the reference period outside of the tropics.

Approximately half of the observed increase in σ_d_ can be accounted for by anthropogenic climate change (Fig. 1e, Supplementary Fig. 2a). The counterfactual simulation produced: 1) σ_d_ trends with a global median of +0.36σ and were spatially similar to that observed, 2) mostly non-significant trends in temperature based departures, and 3) trends in moisture based departures that were approximately a third the magnitude of observed trends. Similarly, excluding the effect of anthropogenic climate change led to a 45% reduction in the extent of global land area with σ_d_ > 2σ (Fig. 1f).

Several factors account for the σ_d_ increase after excluding the modeled influence of anthropogenic climate change, including changes in climate variability and divergence between observed and modeled changes in climate. The sensitivity experiment that excluded trends in annual temperature, precipitation, and reference evapotranspiration had σ_d_ trends that were approximately a third of those observed and trends in climate departures for individual variables that were less than 30% of those observed (Fig. 1e, Supplementary Fig. 1c). By contrast, the sensitivity experiment that excluded trends in annual precipitation had nominal influence in attenuating observed trends in climate departures thus highlighting the importance of changes in temperature and evaporative demand (Supplementary Fig. 1b). Our results further suggest that most of the observed increase in climate departures have materialized through changes in mean climate conditions rather than changes in climate variability. Changes in the interannual variability of individual variables showed confounding signals spatially and across variables (*Supplemental Methods*; Supplementary Table 2). The most notable and widespread change was a global median 15% reduction in the standard deviation of T_n,min_ during 1958–2017. The larger fraction of trends accounted for by explicitly detrending observations versus those using the counterfactual model is expected given that observed σ_d_ trends include changes arising from internal variability.

Lastly, the magnitude of observed changes in climate departures exceeded those estimated from natural variability in a stationary climate. A null model based on 500-years of climate model output run under pre-industrial control simulations^38^ yielded a global median σ_d_ trend comparable to that of the detrended sensitivity experiment (average trend +0.24σ over 60-years, maximum of +0.29σ; Supplementary Fig. 6). Internal decadal variability along with the use of a reference period from which climate departures are calculated and associated sampling biases generally results in positive σ_d_ trends even in a stationary climate. However, the substantially larger magnitude of observed changes versus those obtained using the null model suggest that the observed increases in climate departures over our study period are unlikely due to internal climate variability.

## Discussion

We demonstrate that annual climate conditions over the past three decades have departed substantially from previous decades, that these climate departures exceed what would be expected from natural climate variability, and that approximately half of the magnitude of these changes can be attributed to anthropogenic forcings. While previous studies have highlighted the importance of multivariate extremes and changes thereof^11,13^, we find that multivariate climate departures assessed at annual time scales have rapidly outpaced univariate departures, highlighting the potential latent impacts imposed by observed change to systems adapted to multiple interacting climate variables. The largest positive σ_d_ trends occurred in regions of historically low variance (e.g., equatorial regions)^8,39,40^, in regions such as southern Europe that have seen large changes in climate trends in the variables considered^41^, and in boreal regions that saw joint increases in AET and D that are orthogonal to the historical covariance of these variables. The growing extent of lands with annual multivariate climate departures>2σ in recent decades complements other studies that have considered univariate departures^42^. While our results were specific to the four variables than span moisture and thermal constraints in a given year, trends in multivariate climate departures may differ as a function of the variables chosen.

Trends in multivariate climate departures were largely accounted for by changes in climate means rather than changes in climate variability. While there is a perception that observed climate change has resulted in heightened variability, observational and modeling studies generally do not support widespread detectable changes to date^43,44^. Nonetheless, while observed changes in variability have generally been small, some studies suggest increased hydroclimatic variability or intensification over the 21st century that would further exacerbate increased climate departures^45–47^.

Our framing of univariate and multivariate departures builds on previous studies^3,39,48^ while emphasizing more nuanced ways in which climate has differed from reference conditions on annual timescales. Only considering changes in mean conditions or changes for multi-decadal timescales can obscure departures that occur annually. This type of interannual variability is critical for assessing climate change impacts. For example, years with concurrent high D and T_x,max_ enable fire activity in flammability-limited forests^19^, while years with high moisture availability (high AET, low D) and low T_x,max_ promote recruitment pulses in water-limited forests^49^. Warm summers accompanied by surface water availability (high T_x,max_, high AET) may impact human health directly through heat stress and indirectly through promoting environmental conditions that favor certain vector-borne diseases^50,51^. Annual climate departures are also relevant to impacts on agricultural systems, land-use planning, and built infrastructure^52,53^.

As the climate becomes increasingly unfamiliar to the flora and fauna at a specific location, organisms, including humans, must adapt to changing local conditions or move to maintain suitable climates^54^. Indeed, Earth’s biota is already adapting to climate change. For example, migration and thermophilization of biotic communities has been observed^55,56^. Additionally, humans are increasingly adopting crop varieties, seed provenances, and horticultural practices from other locations with climates that are anticipated to resemble future conditions^3,57^. Unfortunately, the adaptive capacity of systems is not necessarily commensurate with their climate change exposure. For example, thermal specialists in the tropics may be uniquely vulnerable to the large climate departures seen in these regions^58,59^. Collectively, our results suggest that annual multivariate climate departures have changed more dramatically in the last 60 years than univariate estimates of departures for individual climate variables.

## Methods

Monthly data from TerraClimate during 1958–2017 were used for our primary analysis^60^. TerraClimate combines high-resolution multi-decadal climatologies from WorldClim^61^ with coarser-scale temporally varying data from reanalyses and CRU Ts4.0^62^ to create 4-km (1/24°) spatial resolution surfaces of monthly first-order climate variables (e.g., temperature, precipitation), as well as surface water balance products such as AET and D. The water balance model is an accounting-based approach that simulates moisture fluxes using a simple snow model and soil water balance approach for a reference vegetation type. This modeling framework has been widely used in hydrological and ecological studies^10,63^. Other gridded datasets provide similar outputs, including those using more sophisticated surface hydrologic models. However, they do not cover the 60-year period of observational record or are of much coarser spatial resolution. Nonetheless, to interrogate the sensitivity of our results with respect to structural uncertainty of climate data sources, we replicate all analyses for the period 1958–2016 using gridded data from the Princeton Global Meteorological Forcing dataset (version 3) at a 0.25° spatial resolution^64^. Results from these data are presented in *Supplemental Methods*.

Four climate metrics were chosen given their established links to factors that influence Earth’s biota including species occurrence and impacts, and their frequent usage in species distribution models^10,29,32,65^. These metrics included: (1) average maximum temperature of the warmest month for the calendar year (T_x,max_), (2) average minimum temperature of the coldest month for the calendar year (T_n,min_), (3) calendar year cumulative AET, and (4) calendar year cumulative D. These four variables provide complementary information pertinent to ecological and agricultural systems. For example, T_n,min_ can be a limiting factor for some species and agriculture and modify overwinter morality rates of some organisms^66,67^, while D and AET provide a reduced set of biologically relevant and physically based variables that account for the concurrent availability of both water and energy important for both ecological and agricultural impacts^29,68^.

We calculated σ_d_ and its univariate equivalent standardized Euclidean distances for each variable during 1958–2017. The first 30-years (1958–1987) were used to define a baseline period that determine both the centroid (i.e. the mean) and covariance structure using principal components analysis to calculate σ_d_ (*Supplemental Methods*). A given 30-year period comprises a sample of a population of climate statistics that may differ from other baseline periods in some regions due to decadal variability^69^. Likewise, anthropogenic climate forcing is evident throughout the observational record limiting the ability to procure a true ‘natural’ reference state. However, for the basis of this study, the earliest 30-years covers a time period when anthropogenic forcing was substantially less than in recent decades.

We used Mahalanobis distances and its standardization based on the Chi distribution to calculate σ_d_. This approach accounts for covariance among variables, the dimensionality of the data (number of variables), and local inter-annual climate variability thus facilitating comparisons across space and variable combinations. Mahalanobis distances were calculated on standardized data (i.e., normal distributions based on means and standard deviation calculated during 1958–1987). This approach assumes variables adhere to a multivariate Gaussian distribution, which may be reasonable for some variables, but could be problematic for zero-bounded data (e.g., D) and for temperature extremes in some portions of the globe. We conducted a sensitivity analysis that used clamped non-parametric distributions to assess whether results were substantially altered by these assumptions (*Supplemental Methods*). The choice of data transformations did not substantially alter the general results of the study, although the truncated nature of the non-parametric distributions reduced overall magnitudes of departures. More advanced multivariate approaches such as copulas may provide additional nuance beyond the linear approaches used herein^70^. Trends for σ_d_ and climate departure for individual variables during 1958–2017 were calculated using Sen-Theil slope estimator and were considered significant using the Mann-Kendall trend test at p < 0.05. Similarly, standard trends using the same approach were calculated on the raw climate variables.

A counterfactual simulation of terrestrial climate was developed that excludes the modeled influence of anthropogenic climate forcing during 1958–2017. The counterfactual simulation removed first-order modeled trends in monthly climate from observed data similar to that of previous studies^71–73^. We used a pattern scaling approach that applies the median response among 23 different climate models to proximate modeled anthropogenic changes (*Supplemental Methods*)^73^. We also considered two sensitivity tests that use detrended observations to decompose changes in climate departures into those associated with trends versus those associated with changes in intraannual-to-interannual variability. One approach removed only annual precipitation trends, while the other approach removed annual trends in temperature, precipitation, and reference evapotranspiration. Detrending was facilitated by calculating a linear least square fit on annual data. This linear fit was subtracted from the observed time series leaving data for 1958 unaltered. By only removing annual trends, we allow monthly trends to differ in a relative sense from annual counterparts. Data for the counterfactual simulation and sensitivity experiments were run through the water balance model to calculate AET and D.

We consider two additional filters in an effort to avoid misinterpreting results in locations of reduced data quality or where the baseline observations were heavily positively-skewed. First, we omit calculations in locations where annual D or AET was 0 for a majority of the years in the baseline period. Second, we used the data quality flags in TerraClimate inherited from CRU TS4.0 to select pixels where at least four stations contributed to monthly precipitation, temperature, and vapor pressure fields for at least 75% of the period of record. Fig. S4 shows the data from Fig. 2 with data poor locations masked out. Summarized timeseries and reported trends for global land surfaces exclude pixels not meeting either of these criteria.

Lastly, we use 500-years (model years 400–899) of pre-industrial control climate simulated from the LENS experiment which uses NCAR’s Community Earth System Model version 1 (CESM1) with CAM5.2 as its atmospheric model^38^ to develop a null model for changes in σ_d_ that result purely from internal climate variability. Monthly output from LENS was subsequently used in the water balance model, albeit at the native spatial resolution of LENS output. We subsequently calculated σ_d_ using non-overlapping moving 30-year blocks over the simulation (e.g., 430–459) and calculated both forward (e.g., 430–489) and backward (e.g., 400–459) linear trends in global terrestrial median σ_d_ and the fraction of land surfaces with σ_d_ > 2σ. The slope of trends calculated from backward samples was inverted. As with observations, trends were calculated using Sen-Theil slope estimator.

## Supplementary information

Supplemental Materials.

## Data Availability

The primary datasets analyzed in the current study are available through the Northwest Knowledge Network data repository at https://climate.northwestknowledge.net/TERRACLIMATE-DATA/.

## References

[1] Harris RMB (2018). Biological responses to the press and pulse of climate trends and extreme events. Nat. Clim. Chang..

[2] Kawecki TJ, Ebert D (2004). Conceptual issues in local adaptation. Ecol. Lett..

[3] Mahony, C. R., Cannon, A. J., Wang, T. & Aitken, S. N. A closer look at novel climates: new methods and insights at continental to landscape scales. *Glob. Chang. Biol*. (2017). 10.1111/gcb.13645 28145063

[4] Flannigan MD, Harrington JB (1988). A study of the relation of meteorological variables to monthly provincial area burned by wildfire in Canada (1953-80). J. Appl. Meteorol..

[5] Piao S (2010). The impacts of climate change on water resources and agriculture in China. Nature.

[6] Duffy PB (2019). Strengthened scientific support for the Endangerment Finding for atmospheric greenhouse gases. Science.

[7] Abatzoglou JT, Williams AP, Barbero R (2019). Global emergence of anthropogenic climate change in fire weather indices. Geophys. Res. Lett..

[8] Mahlstein I, Knutti R, Solomon S, Portmann RW (2011). Early onset of significant local warming in low latitude countries. Environ. Res. Lett..

[9] Frame D, Joshi M, Hawkins E, Harrington LJ, de Roiste M (2017). Population-based emergence of unfamiliar climates. Nat. Clim. Chang..

[10] Dobrowski SZ (2013). The climate velocity of the contiguous United States during the 20th century. Glob. Chang. Biol..

[11] Hao Z, AghaKouchak A, Phillips TJ (2013). Changes in concurrent monthly precipitation and temperature extremes. Environ. Res. Lett..

[12] Mazdiyasni O, AghaKouchak A (2015). Substantial increase in concurrent droughts and heatwaves in the United States. Proc. Natl. Acad. Sci..

[13] Sarhadi A, Ausín MC, Wiper MP, Touma D, Diffenbaugh NS (2018). Multidimensional risk in a nonstationary climate: Joint probability of increasingly severe warm and dry conditions. Sci. Adv..

[14] Zhou S, Zhang Y, Williams AP, Gentine P (2019). Projected increases in intensity, frequency, and terrestrial carbon costs of compound drought and aridity events. Sci. Adv..

[15] Mora C (2018). Broad threat to humanity from cumulative climate hazards intensified by greenhouse gas emissions. Nat. Clim. Chang..

[16] Trenberth, K. E. & Shea, D. J. Relationships between precipitation and surface temperature. *Geophys. Res. Lett*. **32** (2005).

[17] Seneviratne SI (2010). Investigating soil moisture–climate interactions in a changing climate: A review. Earth-Science Rev..

[18] Willmott CJ, Rowe CM, Mintz Y (1985). Climatology of the terrestrial seasonal water cycle. J. Climatol..

[19] Abatzoglou JT, Williams AP, Boschetti L, Zubkova M, Kolden CA (2018). Global patterns of interannual climate-fire relationships. Glob. Chang. Biol..

[20] Allen CD (2010). A global overview of drought and heat-induced tree mortality reveals emerging climate change risks for forests. For. Ecol. Manage..

[21] van Mantgem PJ (2013). Climatic stress increases forest fire severity across the western U nited S tates. Ecol. Lett..

[22] Wada Y (2013). Multimodel projections and uncertainties of irrigation water demand under climate change. Geophys. Res. Lett..

[23] Zscheischler J, Seneviratne SI (2017). Dependence of drivers affects risks associated with compound events. Sci. Adv..

[24] Burke M, Hsiang SM, Miguel E (2015). Global non-linear effect of temperature on economic production. Nature.

[25] Schlenker W, Roberts MJ (2009). Nonlinear temperature effects indicate severe damages to US crop yields under climate change. Proc. Natl. Acad. Sci..

[26] Inouye DW (2000). The ecological and evolutionary significance of frost in the context of climate change. Ecol. Lett..

[27] Apadula F, Bassini A, Elli A, Scapin S (2012). Relationships between meteorological variables and monthly electricity demand. Appl. Energy.

[28] Deryng D, Conway D, Ramankutty N, Price J, Warren R (2014). Global crop yield response to extreme heat stress under multiple climate change futures. Environ. Res. Lett..

[29] Stephenson Nathan L. (1990). Climatic Control of Vegetation Distribution: The Role of the Water Balance. The American Naturalist.

[30] Taylor PG (2017). Temperature and rainfall interact to control carbon cycling in tropical forests. Ecol. Lett..

[31] Anderegg WRL (2015). Pervasive drought legacies in forest ecosystems and their implications for carbon cycle models. Science.

[32] Lutz JA, van Wagtendonk JW, Franklin JF (2010). Climatic water deficit, tree species ranges, and climate change in Yosemite National Park. J. Biogeogr..

[33] Parks SA, Parisien M-A, Miller C, Dobrowski SZ (2014). Fire activity and severity in the western US vary along proxy gradients representing fuel amount and fuel moisture. Plos One.

[34] Westerling AL, Turner MG, Smithwick EAH, Romme WH, Ryan MG (2011). Continued warming could transform Greater Yellowstone fire regimes by mid-21st century. Proc. Natl. Acad. Sci. USA.

[35] Jackson ST, Betancourt JL, Booth RK, Gray ST (2009). Ecology and the ratchet of events: Climate variability, niche dimensions, and species distributions. Proc. Natl. Acad. Sci..

[36] Fitzpatrick MC, Dunn RR (2019). Contemporary climatic analogs for 540 North American urban areas in the late 21st century. Nat. Commun..

[37] Wolter K, Timlin MS (2011). El Niño/Southern Oscillation behaviour since 1871 as diagnosed in an extended multivariate ENSO index (MEI. ext). Int. J. Climatol..

[38] Kay JE (2015). The Community Earth System Model (CESM) large ensemble project: A community resource for studying climate change in the presence of internal climate variability. Bull. Am. Meteorol. Soc..

[39] Mora C (2013). The projected timing of climate departure from recent variability. Nature.

[40] Hawkins, E. & Sutton, R. Time of emergence of climate signals. *Geophys. Res. Lett*. **39**, (2012).

[41] Cook BI, Anchukaitis KJ, Touchan R, Meko DM, Cook ER (2016). Spatiotemporal drought variability in the Mediterranean over the last 900 years. J. Geophys. Res. Atmos..

[42] Coumou D, Robinson A (2013). Historic and future increase in the global land area affected by monthly heat extremes. Environ. Res. Lett..

[43] Huntingford C, Jones PD, Livina VN, Lenton TM, Cox PM (2013). No increase in global temperature variability despite changing regional patterns. Nature.

[44] Screen JA (2014). Arctic amplification decreases temperature variance in northern mid-to high-latitudes. Nat. Clim. Chang..

[45] Swain DL, Langenbrunner B, Neelin JD, Hall A (2018). Increasing precipitation volatility in twenty-first-century California. Nat. Clim. Chang..

[46] Diffenbaugh, N. S. & Ashfaq, M. Intensification of hot extremes in the United States. *Geophys. Res. Lett*. **37**, (2010).

[47] Seager R, Naik N, Vogel L (2012). Does global warming cause intensified interannual hydroclimate variability?. J. Clim..

[48] Mahony CR, Cannon AJ (2018). Wetter summers can intensify departures from natural variability in a warming climate. Nat. Commun..

[49] Davis KT (2019). Wildfires and climate change push low-elevation forests across a critical climate threshold for tree regeneration. Proc. Natl. Acad. Sci..

[50] Sherwood SC, Huber M (2010). An adaptability limit to climate change due to heat stress. Proc. Natl. Acad. Sci..

[51] Ebi KL, Nealon J (2016). Dengue in a changing climate. Environ. Res..

[52] Hatfield, J. *et al*. Climate change impacts in the United States: The third national climate assessment. *Washington, DC* 150–174 (2014).

[53] Sivakumar, M. V. K., Das, H. P. & Brunini, O. Impacts of present and future climate variability and change on agriculture and forestry in the arid and semi-arid tropics. in *increasing climate variability and change* 31–72 (Springer, 2005).

[54] Parmesan C (2006). Ecological and evolutionary responses to recent climate change. Annu. Rev. Ecol. Evol. Syst..

[55] Chen I-C, Hill JK, Ohlemüller R, Roy DB, Thomas CD (2011). Rapid range shifts of species associated with high levels of climate warming. Science.

[56] Bertrand R (2011). Changes in plant community composition lag behind climate warming in lowland forests. Nature.

[57] Thomas CD (2011). Translocation of species, climate change, and the end of trying to recreate past ecological communities. Trends Ecol. Evol..

[58] Tewksbury JJ, Huey RB, Deutsch CA (2008). Putting the heat on tropical animals. Science.

[59] Huey RB (2012). Predicting organismal vulnerability to climate warming: roles of behaviour, physiology and adaptation. Philos. Trans. R. Soc. B Biol. Sci..

[60] Abatzoglou JT, Dobrowski SZ, Parks SA, Hegewisch KC (2018). TerraClimate, a high-resolution global dataset of monthly climate and climatic water balance from 1958–2015. Sci. Data.

[61] Fick Stephen E., Hijmans Robert J. (2017). WorldClim 2: new 1-km spatial resolution climate surfaces for global land areas. International Journal of Climatology.

[62] Harris I, Jones PD, Osborn TJ, Lister DH (2014). Updated high-resolution grids of monthly climatic observations – the CRU TS3.10 Dataset. Int. J. Climatol..

[63] Gleick PH (1987). The development and testing of a water balance model for climate impact assessment: modeling the Sacramento basin. Water Resour. Res..

[64] Sheffield J, Goteti G, Wood EF (2006). Development of a 50-year high-resolution global dataset of meteorological forcings for land surface modeling. J. Clim..

[65] Parks SA (2015). Wildland fire deficit and surplus in the western United States, 1984–2012. Ecosphere.

[66] Parker LE, Abatzoglou JT (2016). Projected changes in cold hardiness zones and suitable overwinter ranges of perennial crops over the United States. Environ. Res. Lett..

[67] Williams CM, Henry HAL, Sinclair BJ (2015). Cold truths: how winter drives responses of terrestrial organisms to climate change. Biol. Rev..

[68] Wriedt G, V der Velde M, Aloe A, Bouraoui F (2009). Estimating irrigation water requirements in Europe. J. Hydrol..

[69] Hulme M, New M (1997). Dependence of large-scale precipitation climatologies on temporal and spatial sampling. J. Clim..

[70] AghaKouchak A, Cheng L, Mazdiyasni O, Farahmand A (2014). Global warming and changes in risk of concurrent climate extremes: Insights from the 2014 California drought. Geophys. Res. Lett..

[71] Abatzoglou JT, Williams AP (2016). Impact of anthropogenic climate change on wildfire across western US forests. Proc. Natl. Acad. Sci..

[72] Williams AP (2015). Contribution of anthropogenic warming to California drought during 2012–2014. Geophys. Res. Lett..

[73] Mitchell TD (2003). Pattern scaling: an examination of the accuracy of the technique for describing future climates. Clim. Change.

