# Supplementary material for: Multivariate climate departures have outpaced univariate changes across global lands
Source: Sci Rep. 2020 Mar 3;10:3891. doi: 10.1038/s41598-020-60270-5 (PMC7054431; doi:10.1038/s41598-020-60270-5)
Supplement: Supplementary file 1 — Supplemental Materials. [file 41598_2020_60270_MOESM1_ESM.docx]

**Supplementary Information for**

**Multivariate climate departures have outpaced univariate changes across global lands**

John T. Abatzoglou^1,2*^, Solomon Z. Dobrowski^3^, Sean A. Parks^4^

^1^Department of Geography

University of Idaho, Moscow

875 Perimeter Dr.

Moscow, ID 83844 USA

jabatzoglou@uidaho.edu

(208) 885-6239

orcid.org/0000-0001-7599-9750

* corresponding author

^2^ Management of Complex Systems Department, University of California, Merced

^3^ University of Montana, Department of Forest Management

^4^ USDA Forest Service, Rocky Mountain Research Station, Aldo Leopold Wilderness Research Institute

**Conceptual presentation of multivariate interannual climate departure**

Interannual anomalies are regularly used in climate summaries and quantify the difference in a given climate variable from a baseline period. Likewise, one can consider the distance of individual climate variables from a baseline period as the absolute value or standardized Euclidean distance from a baseline period. Consider a time series of the annual AET total during 1958-1997 along the western slope of the Cascade mountains in Washington State, US (47°N, 122°W; Fig S7a). We impose synthetic changes in these data to reflect (a) a 50% increase in the standard deviation with no change in the mean, and (b) a 25-mm increase in the mean (equivalent to +0.71σ) with no change in variability (Fig. S7b). Both synthetic changes increase departures from baseline climate average, but standard approaches for examining changes (e.g., changes in means) fail to capture distributional changes that may impact climate sensitive systems.

The magnitude of departures from baseline conditions is further confounded in multivariate space given the covariance structure between climate variables. We extend our example by incorporating annual D and annual AET data (Fig. S7c). There is a strong negative correlation between AET and D on interannual timescales as is common for most land surfaces that are moisture-limited. The strong covariance between these data suggest that it is inappropriate to treat the data independently through standardized Euclidean distances. The Mahalanobis distance is used to compactly synthesize multiple variables and account for their covariance structure and measures the distance in multivariate space away from a centroid through principal components analysis of standardized anomalies. Mahalanobis distances are transformed to standardized distances using the Chi distribution, σ_d_ ^3^ that we refer to as climate departures. The chi distribution is the probability density function of the non-squared Mahalanobis distances from multivariate normal observations to their mean that explicitly accounts for the number of variables used. σ_d_ can be considered a multivariate z-score and interpretable based on the standard normal distribution.

We contrast three hypothetical imposed changes in climate to our reference timeseries to illustrate the novelty of applying the concept of σ_d_ to interannual timescales. First, we impose a 50% increase in the standard deviation of both AET and D (Fig. S7d). The resultant data have the same mean, but heightened variability, resulting in an overall increase in σ_d_ of +0.62σ. The second and third hypothetical scenarios impose uniform increases in AET of +25mm with no change in variability. Scenario two imposes an increase in D by 30mm that is orthogonal to internal AET-D variability (Fig S7e), while scenario three imposes a decrease in D by 30mm that is parallel to AET-D variability (Fig S7f). Approaches that quantify multivariate distances through Euclidean approaches would yield no difference between scenarios two and three ^73^. However, substantial differences in σ_d_ are evident for the same change in AET and same magnitude change in D. Notably, substantially smaller changes in σ_d_ occur when changes in bivariate climate space follow the trajectory of historic interannual variability (+0.16σ), compared to cases where changes in bivariate climate space oppose interannual variability (+0.92σ). While all three cases of imposed changes may have impacts, we focus our efforts on this multivariate measure of climate departure as it represents a generalizable way to encapsulate departures from a reference climate state.

**Pattern scaling approach for counterfactual simulations**

A pattern scaling approach is used to construct a representative approximation of first-order changes in climate in response to anthropogenic forcing. Pattern scaling uses the concept that geographic patterns of climate change scale quasi-linearly with changes in global mean temperature ^74^. Patterns are developed through linear regression between changes in local climate (e.g., monthly temperature) and changes in global mean annual temperature relative to a reference period.

Pattern scalings are developed for monthly climate variables of maximum temperature, minimum temperature, specific humidity, 10-m wind speed, precipitation, and surface downward shortwave radiation. We acquired climate projections from 23 CMIP5 climate models (Table S3) for two 30-year time periods, a quasi pre-industrial period (1850-1879) using historical forcing and an end of the 21^st^ century period (2070-2099) using the RCP8.5 forcing. Regression coefficients were calculated separately for each month and each variable using the changes in climate between the two 30-year periods. Differences for all variables except precipitation are treated as absolute changes. For precipitation, we considered relative changes (i.e., absolute change divided by preindustrial average). Pattern scaling was calculated separately for each model; but herein we used the 23-model median. We note that pattern scaling as applied here does not consider any changes in variability as simulated by models.

Pattern scalings are multiplied by changes in global mean annual temperature from a baseline 1958-1987 period. We additionally applied a moving 11-year average to modeled estimates to avoid entraining model forcing from solar variability or volcanic eruptions. These values were then removed from the observed data in an additive sense for all variables, except for precipitation which was done in a multiplicative sense.

**Sensitivity to data transformation**

To address the potential limitations of the Mahalanobis distance approach and its parametric assumptions, we perform a sensitivity analysis using a truncated nonparametric distribution. We used a generalizable approach that uses data rank as a means of mapping data to a normal distribution ^75^. Analogous to standardized anomalies developed relative to a baseline period, the distributions for this non-parametric transform are developed for the baseline period (1958-1987) and applied over the 1958-2017. We clamp data to the range of the baseline period, which results in a conservative approach that does not allow for novel conditions. For comparison, applying this transform to the example in Figure S7 leads to reductions in increased σ: +0.4, +0.74, and +0.05 were seen for scenarios in Fig S7d, S7e, and S7f, respectively.

Results shown in Figure S1 are qualitatively similar to those of Figure 1. However, the magnitude of changes was reduced moderately for all variables. The global median σ_d_ trend was +0.6σ during 1958-2017, approximately a quarter less than that using parametric approaches. Similar proportional reductions were seen in trends in climate departures for individual variables. However, the primary interpretation remained unchanged, namely that changes were substantially stronger for multivariate measures than univariate approaches.

**Sensitivity to structural uncertainty in underlying climate data**

Structural uncertainty in climate data can be problematic for the interpretation of climate variability and trends. The TerraClimate data used in our main analysis includes spatiotemporal metadata on data quality inherited from parent datasets of CRUTs4.0. As such, there are portions of the globe with limited station-based observations that may be prone to heightened uncertainty through time which can affect analyses of trends. We repeated calculations using version 3 of the Princeton Gridded Meteorological (PGM) data ^64^ which contained the requisite variables for calculating reference evapotranspiration using the Penman-Monteith approach and running water balance calculations and covered the time period of interest (1958-2016, data for 2017 were not available at the time of analysis).

Results show similar overall trends in climate departures to those calculated from TerraClimate. Overall, the global terrestrial median trend in σ_d_ was 0.84σ over the 59-year record, comparable to estimates using TerraClimate (Fig. S9). The largest differences were seen in trends for distance in T_n,min_ and D across central and northern Africa where data sparseness is widely known to result in uncertainties across observational datasets and omitted in summaries highlighted in Figure 1. Some differences in trends in climate departure for individual variables were evident with the PGM data showing larger trends in T_n,min_ departures than TerraClimate, but reduced trends for T_x,max_ and D departures. Given the overall similarity to macroscale global trends, we suggest differences in datasets are not a substantial source of uncertainty for this analysis.

**Assessment of changing variability**

Linear trends in variability for the four annual climate variables were calculated by using 11-year moving windows of standard deviation ^43^. We further normalize variability by dividing each time series by the standard deviation calculated using the first 30-years of the observational record. Linear trends were calculated using linear least squares regression and results are considered significant at p<0.05 after accounting for serial correlation effects in the time series ^76^.


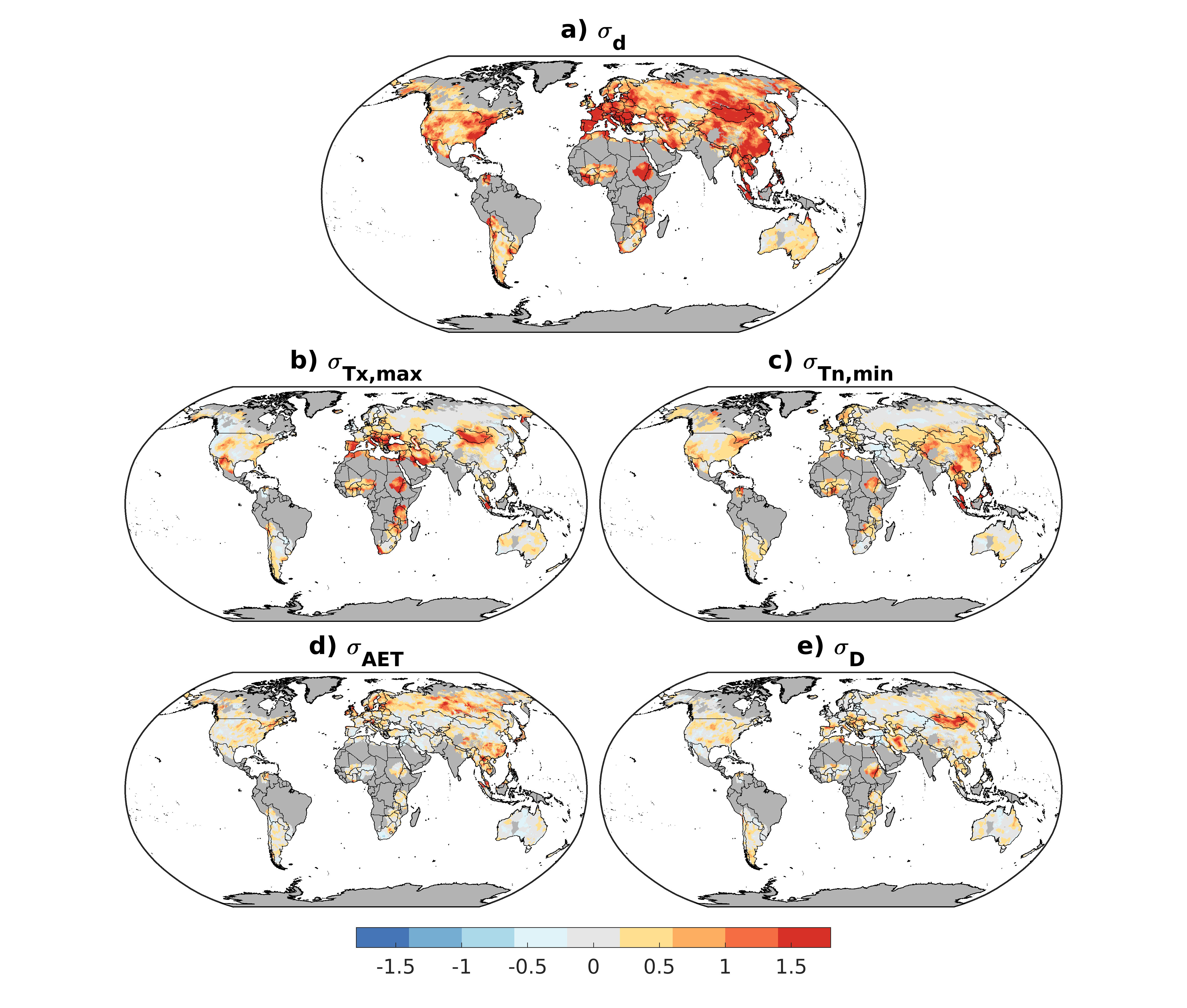


**Supplementary** **Figure 1:** As for Figure 2, but masking out in grey all land areas not meeting data quality standards specified in the text. Only areas meeting data quality standards were used in summary statistics provided.


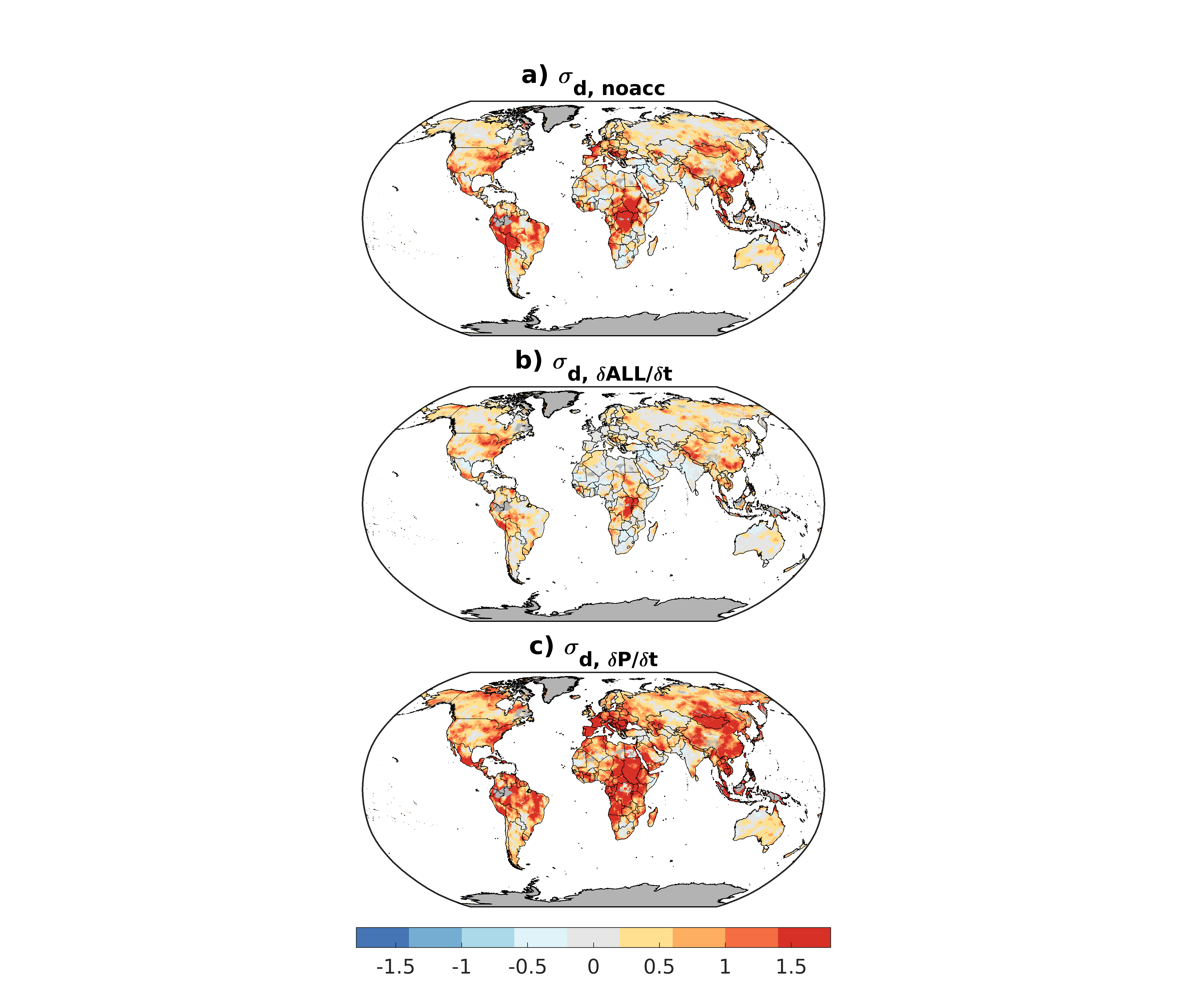


**Supplementary** **Figure 2:** Comparison of trends in multivariate climate departure for (a) counterfactual and climate sensitivity experiments using (b) detrended annual precipitation, temperature, and reference evapotranspiration, and (c) detrended annual precipitation. Land areas where annual D or AET was 0 for a majority of the years during 1958-1987 are shown in grey.


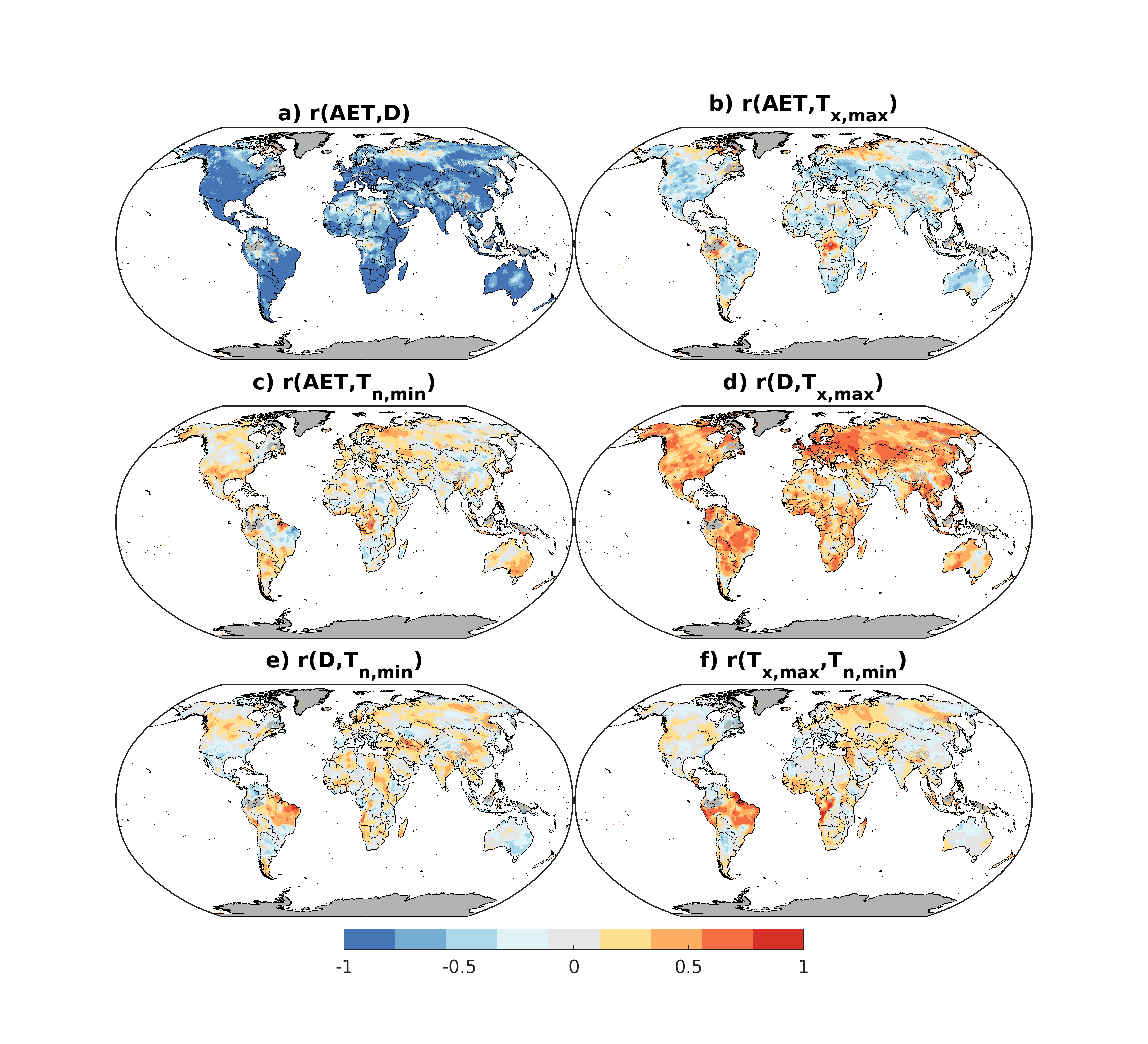


**Supplementary** **Figure 3:** Correlation across variables during reference period 1958-1987. Land areas where annual D or AET was 0 for a majority of the years during 1958-1987 are shown in grey.


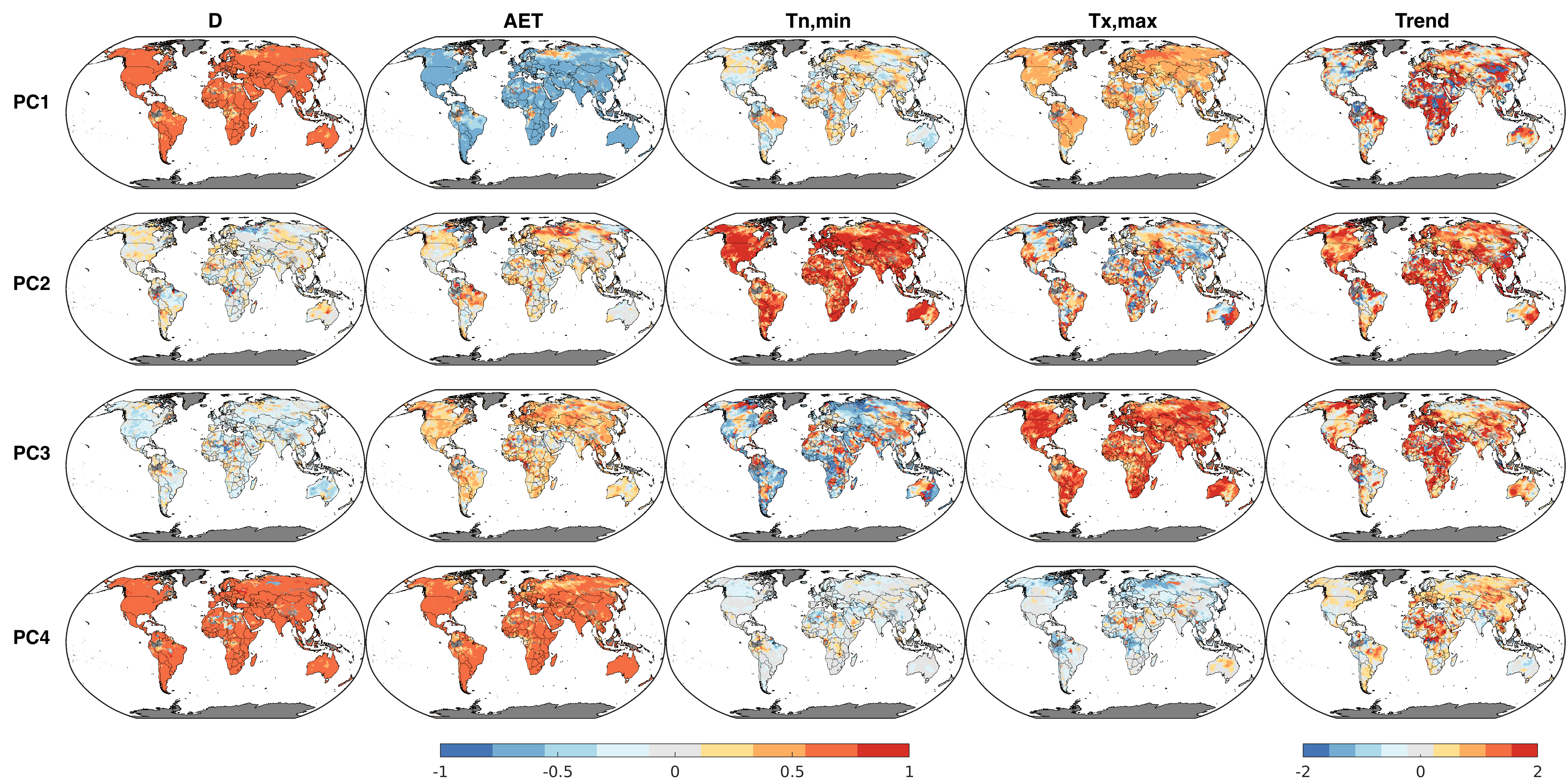


**Supplementary** **Figure 4:** Loading pattern for PC’s 1 (top) to 4 (bottom) to the four different variables of interest (columns 1-4). The right hand panel shows the linear least squares trend in the PC score in units of standard deviations over the 60-yr period 1958-2017.


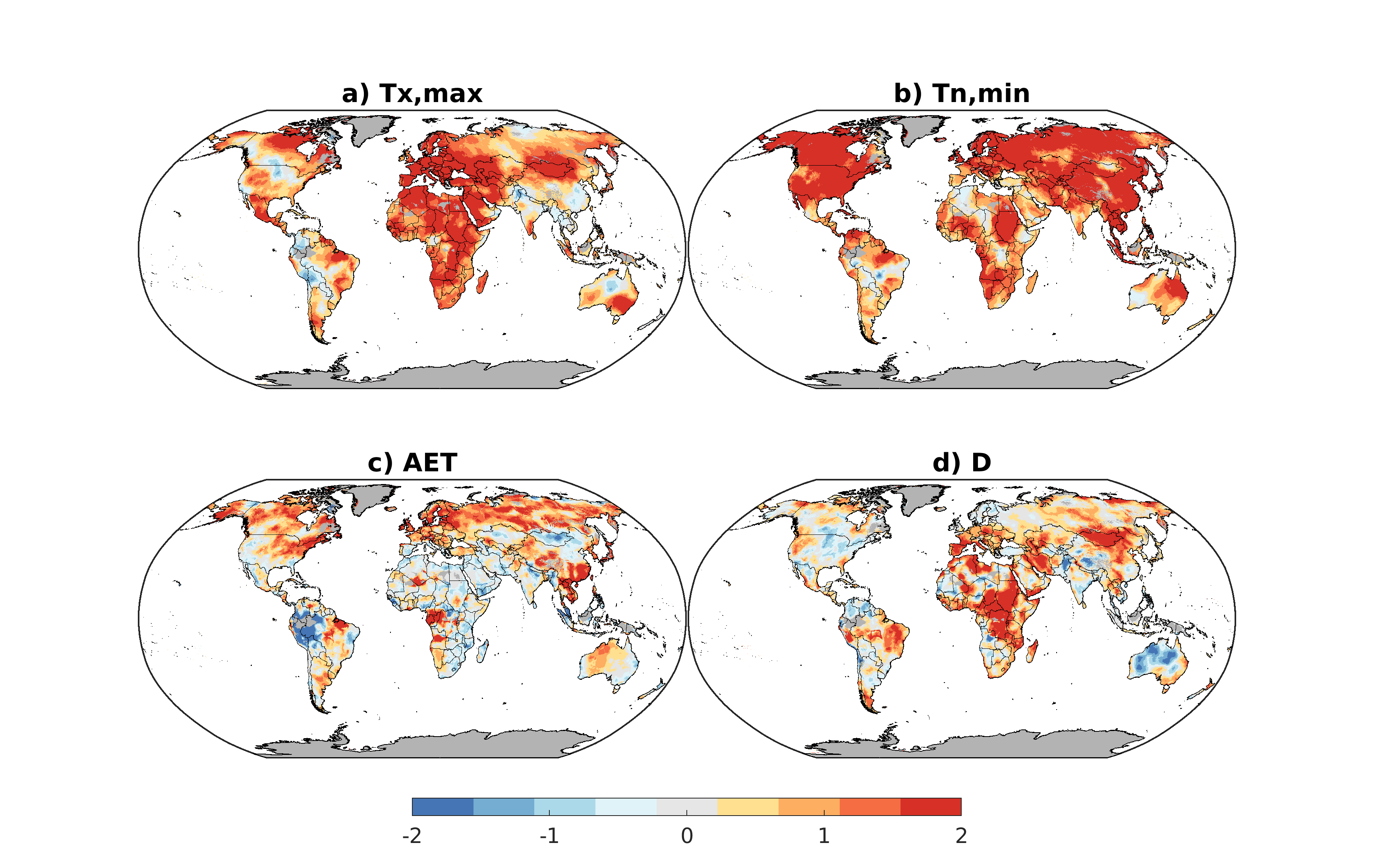


**Supplementary** **Figure 5:** Linear trends (1958-2017) in the four variables examined. Trends are expressed in standardized units over the 60-year period. Land areas where annual D or AET was 0 for a majority of the years in the baseline period are shown in grey.


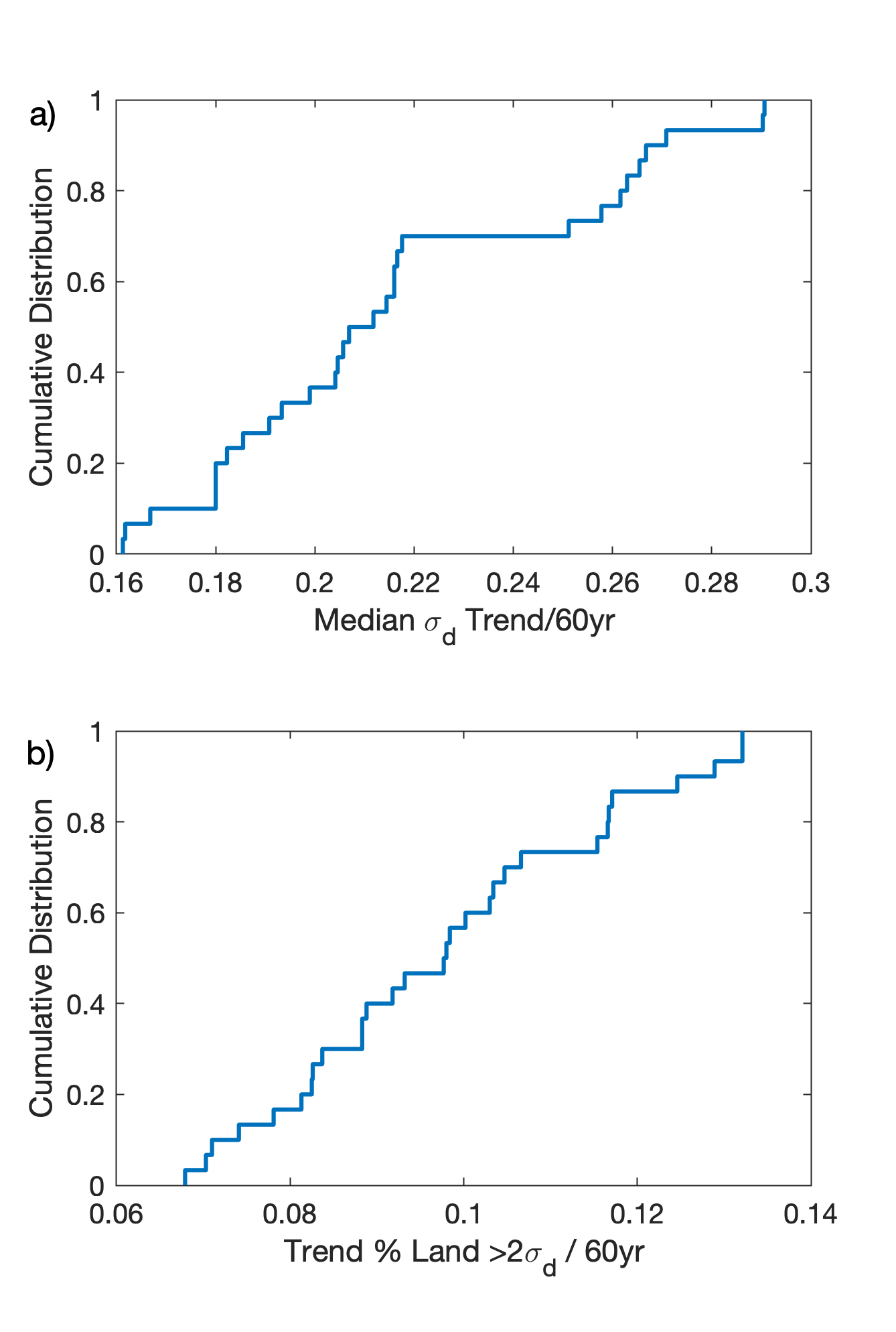


**Supplementary** **Figure 6:** Cumulative distribution of (a) 60-yr trend in median multivariate climate departure of global land areas, and (b) 60-yr trend in percent of global land surface with multivariate climate departure exceeding 2 standard deviations from LENS experiments. Results were taken from a 500-year control simulation from LENS and differences were examined between 60-year block samples using the first 30-years to define reference conditions.


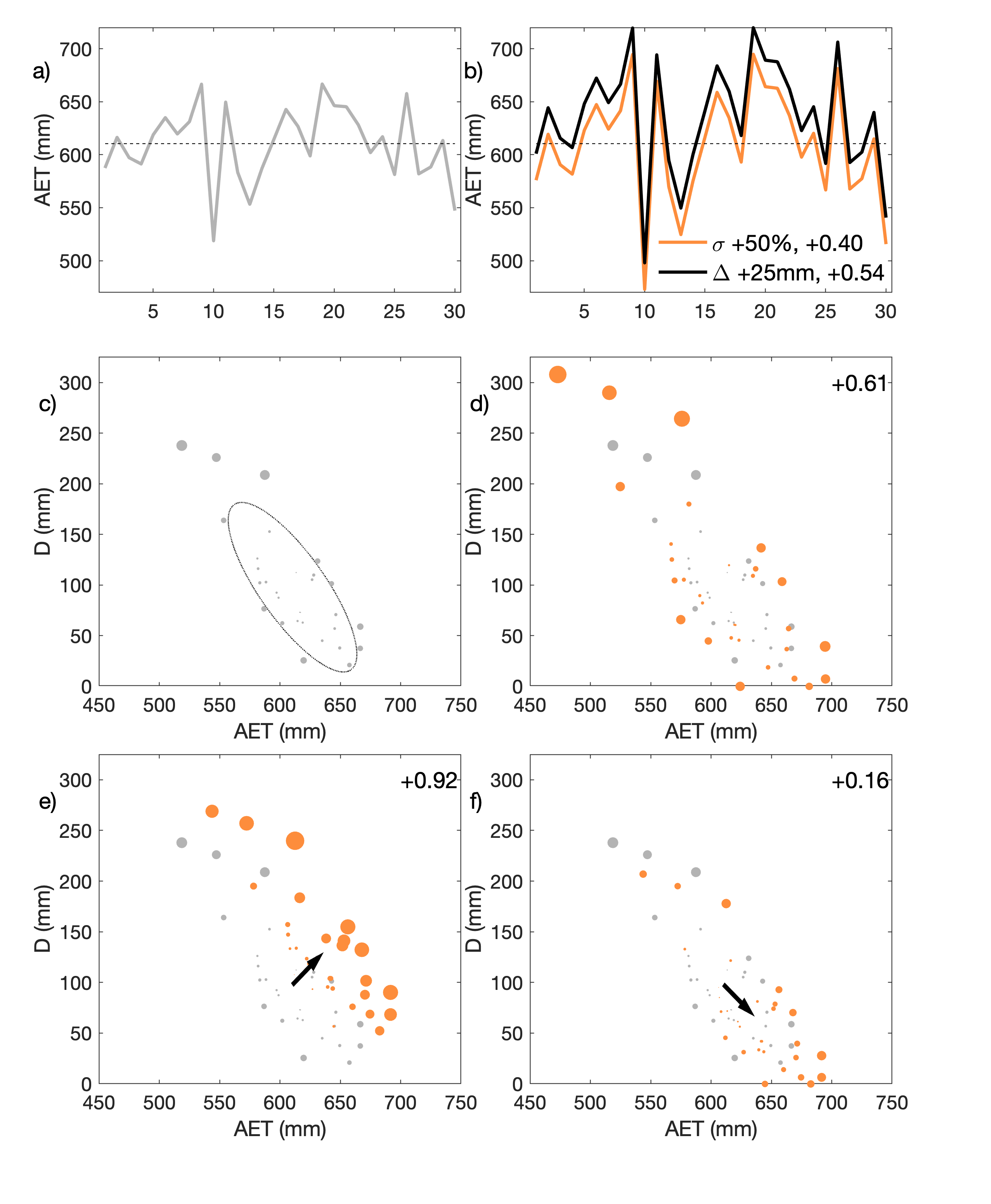


**Supplementary** **Figure 7:** Hypothetical example of multivariate climate departure. Panel (a) shows a time series of annual actual evapotranspiration (AET) taken from western Washington (47°N, 122°W) for 1958-1987, with the dashed horizontal line showing the average for this baseline period. Panel (b) shows the same time series with a 50% increase in variability (orange) and a +25mm increase in the mean (black). (c) Bivariate relationship between and climatic water deficit (D) and AET (grey dots), showing the strong negative correlation between variables and the ellipse shows the 1-sigma isoline of the Mahalanobis distance. The area of each dot represents its multivariate climate departure. Panel (d) overlays the same 30-year climate data where the standard deviation of D and AET increased by 50% without any change in the mean (orange dots). Panel (e) shows results for no change in variance with a 25mm change in mean AET with commensurate increase in D orthogonal to the interannual variability, with the black arrow showing the overall change in the mean. Panel (f) shows results for no change in variance with a 25mm increase in mean AET and with commensurate decrease in D parallel to interannual variability. For reference, the mean difference in Mahalanobis distance between the baseline and simulated data is provided in the upper-righthand corner of each plot.


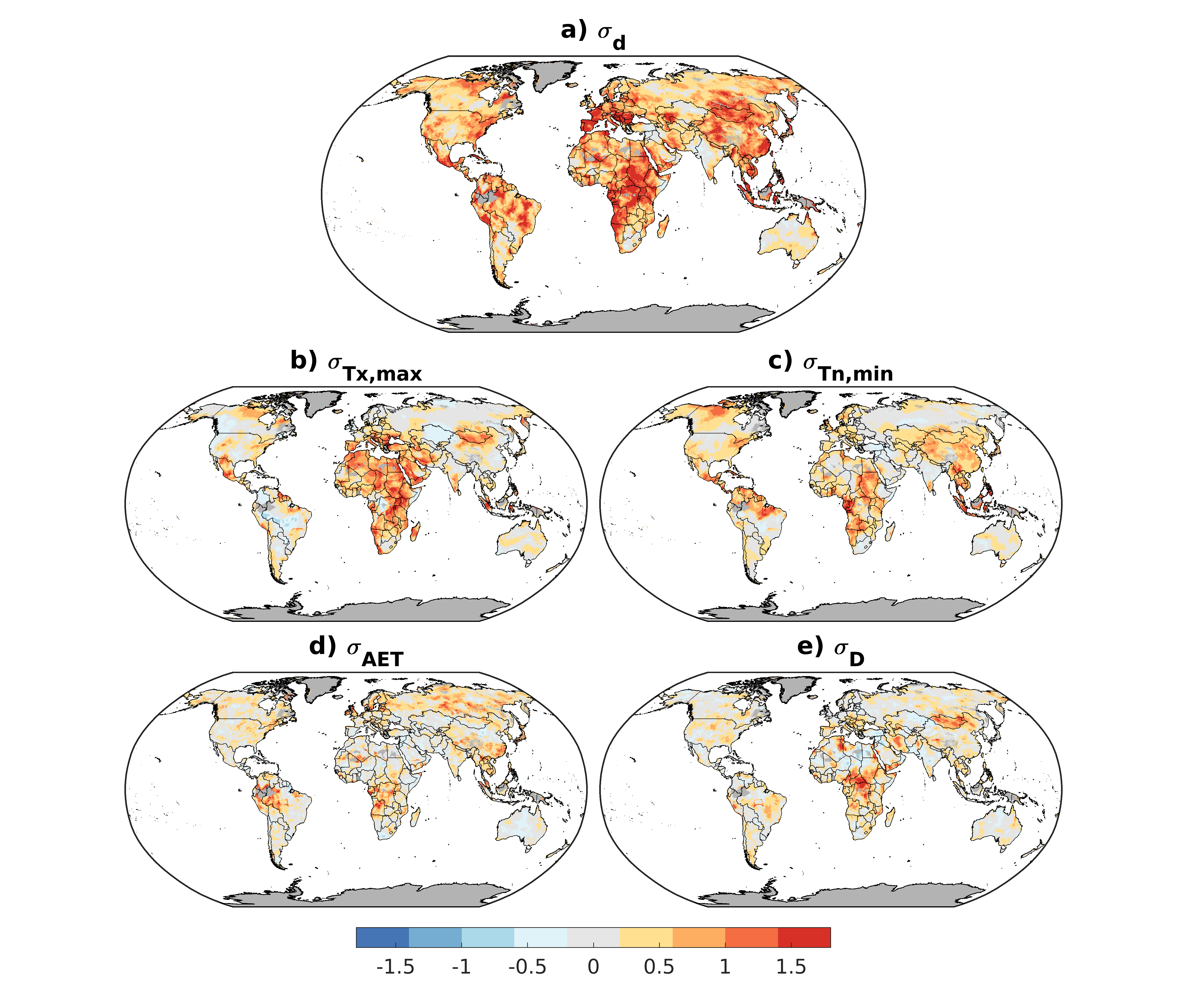


**Supplementary** **Figure 8:** As for Figure 2 using nonparametric standardization procedures. Note the relative reduction in magnitude of change across all variables.


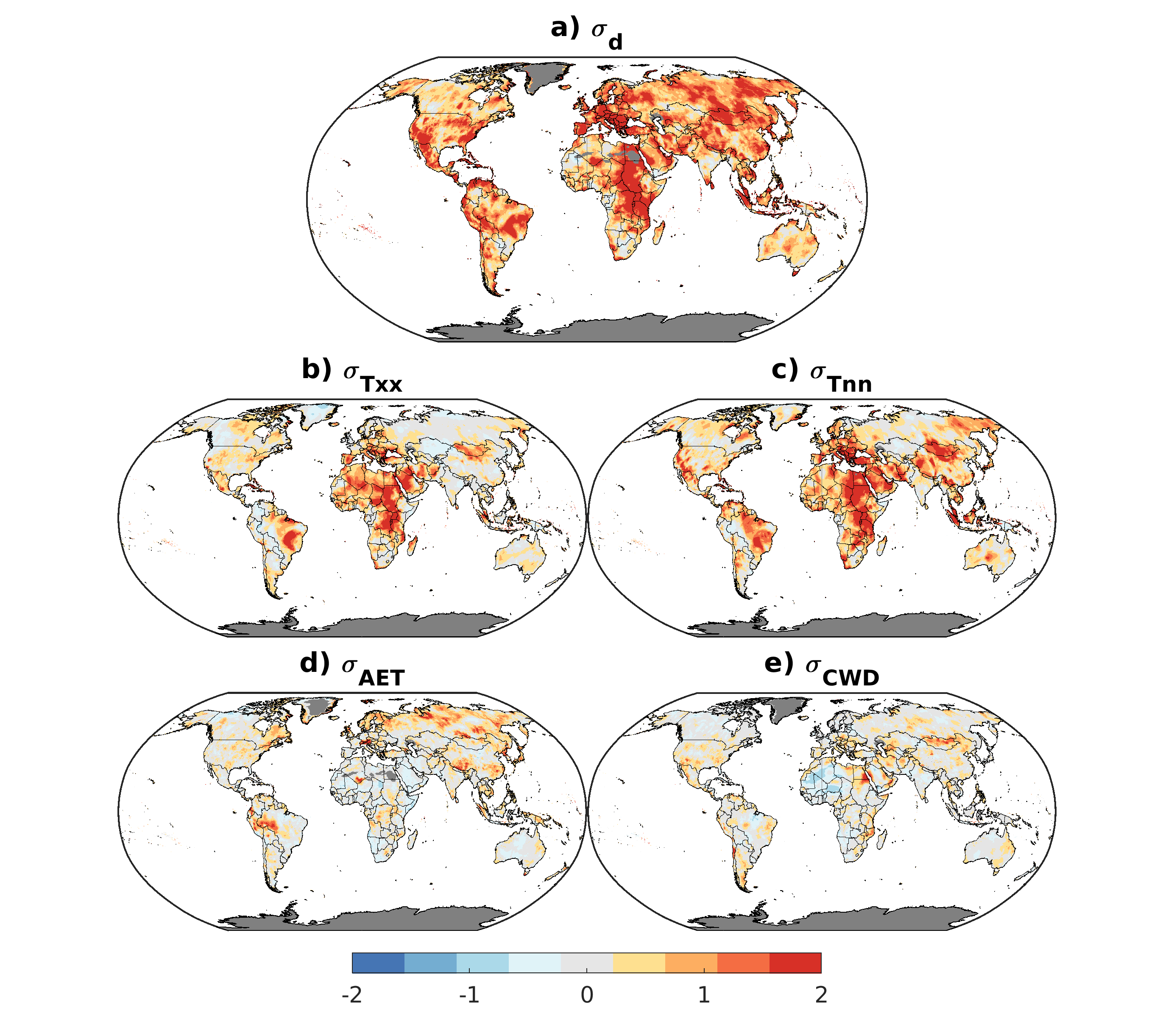


**Supplementary** **Figure 9:** As for Figure 2, but using data from Sheffield et al., (2006) for 1958-2016.

|  | PC1 | PC2 | PC3 | PC4 |
| --- | --- | --- | --- | --- |
| Fractional area where exclusion of individual PC accounts for a majority of significant positive trend | 1.1% | 9.2% | 16.1% | 22.0% |
| Median sigma trend during 1958-2017 | 0.96 | 0.80 | 0.71 | 0.68 |

**Supplementary** **Table 1:** Sensitivity analysis of trends in each of the principal components (PC). The top row shows the percent of global land areas with significant positive σ_d_ trends where excluding trends for including PC reduced the overall σ_d_ by at least 50%. The bottom row reports the median trend in each PC over the 1958-2017 period.

|  | T_x,max_ | T_n,min_ | AET | D |
| --- | --- | --- | --- | --- |
| Significant Increase Fractional area | 10% | 4% | 10% | 10% |
| Significant Decrease Fractional area | 5% | 14% | 6% | 6% |
| Median trend (% relative to 1958-1987) | 8% | -15% | 10% | 8% |

**Supplementary** **Table 2:** Fractional coverage of terrestrial surfaces with statistically significant increases and decreases in standard deviation of the four different climate metrics during 1958-2017. Bottom row shows global median trend expressed in normalized variance units per 60 years (variance is normalized by the 1958-1987 period).

**Supplementary Table 3:** 23 CMIP5 climate models used for developing scaling factors.

| **Model Name** | **Model Agency and Country** | **Ensemble Used** |
| --- | --- | --- |
| ACCESS1-0 | Commonwealth Scientific and Industrial Research Organization (CSIRO) and Bureau of Meteorology (BOM) (Australia) | ri1ip1 |
| ACCESS1-3 | Commonwealth Scientific and Industrial Research Organization (CSIRO) and Bureau of Meteorology (BOM) (Australia) | ri1ip1 |
| bcc-csm1-1 | Beijing Climate Center; China Meteorological Administration (China) | r1i1p1 |
| bcc-csm1-1-m | Beijing Climate Center; China Meteorological Administration (China) | r1i1p1 |
| CanESM2 | Canadian Centre for Climate Modeling and Analysis (Canada) | r1i1p1 |
| CESM1-CAM5 | Community Earth System Model Contributors (United States) | ri1ip1 |
| CNRM-CM5 | National Centre of Meteorological Research (France) | r1i1p1 |
| CSIRO-Mk3-6-0 | Commonwealth Scientific and Industrial Research Organization/Queensland Climate Change Centre of Excellence (Australia) | r1i1p1 |
| GFDL-CM3 | NOAA Geophysical Fluid Dynamics Laboratory (United States) | ri1ip1 |
| GFDL-ESM2M | NOAA Geophysical Fluid Dynamics Laboratory (United States) | r1i1p1 |
| GFDL-ESM2G | NOAA Geophysical Fluid Dynamics Laboratory (United States) | r1i1p1 |
| GISS-E2-H | NASA Goddard Institute for Space Studies (United States) | ri1ip1 |
| GISS-E2-R | NASA Goddard Institute for Space Studies (United States) | ri1ip1 |
| HadGEM2-ES | Met Office Hadley Center (United Kingdom) | r1i1p1 |
| HadGEM2-CC | Met Office Hadley Center (United Kingdom) | r1i1p1 |
| inmcm4 | Institute for Numerical Mathematics (Russia) | r1i1p1 |
| IPSL-CM5A-LR | Institut Pierre Simon Laplace (France) | r1i1p1 |
| IPSL-CM5A-MR | Institut Pierre Simon Laplace (France) | r1i1p1 |
| IPSL-CM5B-LR | Institut Pierre Simon Laplace (France) | r1i1p1 |
| MIROC5 | Atmosphere and Ocean Research Institute, The University of Tokyo; National Institute for Environmental Studies; Japan Agency for Marine-Earth Science and Technology (Japan) | r1i1p1 |
| MIROC-ESM | Japan Agency for Marine-Earth Science and Technology; Atmosphere and Ocean Research Institute, The University of Tokyo; National Institute for Environmental Studies (Japan) | r1i1p1 |
| MIROC-ESM-CHEM | Japan Agency for Marine-Earth Science and Technology; Atmosphere and Ocean Research Institute, The University of Tokyo; National Institute for Environmental Studies (Japan) | r1i1p1 |
| MRI-CGCM3 | Meteorological Research Institute (Japan) | r1i1p1 |
